# A Fully Integrated, Power-Efficient, 0.07–2.08 mA, High-Voltage Neural Stimulator in a Standard CMOS Process

**DOI:** 10.3390/s22176429

**Published:** 2022-08-26

**Authors:** David Palomeque-Mangut, Ángel Rodríguez-Vázquez, Manuel Delgado-Restituto

**Affiliations:** Seville Institute of Microelectronics (CSIC-US), 41092 Sevilla, Spain

**Keywords:** neural stimulator, neuromodulation, neural implant, high voltage compliance, DC-DC converter, charge pump, CMOS, stacked transistors, dynamic gate biasing

## Abstract

This paper presents a fully integrated high-voltage (HV) neural stimulator with on-chip HV generation. It consists of a neural stimulator front-end that delivers stimulation currents up to 2.08 mA with 5 bits resolution and a switched-capacitor DC-DC converter that generates a programmable voltage supply from 4.2 V to 13.2 V with 4 bits resolution. The solution was designed and fabricated in a standard 180 nm 1.8 V/3.3 V CMOS process and occupied an active area of 2.34 mm${}^{2}$. Circuit-level and block-level techniques, such as a proposed high-compliance voltage cell, have been used for implementing HV circuits in a low-voltage CMOS process. Experimental validation with an electrical model of the electrode–tissue interface showed that (1) the neural stimulator can handle voltage supplies up to 4 times higher than the technology’s nominal supply, (2) residual charge—without passive discharging phase—was below 0.12% for the whole range of stimulation currents, (3) a stimulation current of 2 mA can be delivered with a voltage drop of 0.9 V, and (4) an overall power efficiency of 48% was obtained at maximum stimulation current.

## 1. Introduction

**Figure 1:**
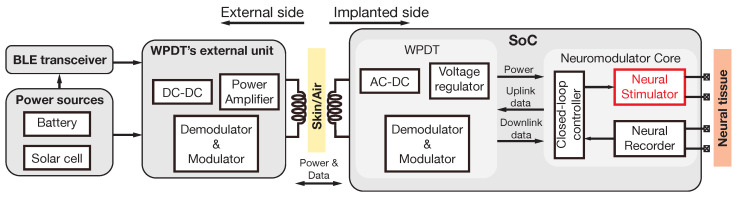
Simplified block diagram of the wirelessly powered neural implant, including power sources, a Bluetooth Low-Energy (BLE) transceiver, Wireless Power and Data Transfer (WPDT) external and internal units, and neuromodulation System on Chip (SoC). The circuit presented in this work is drawn in red.

Neural implants for the treatment of disorders such as Parkinson’s disease, essential tremor, dystonia, epilepsy, chrohin pain, and others [1,2,3,4] generally comprise two main functionalities: neural stimulation and neural recording [5,6]. As an illustration, Figure 1 shows the simplified block diagram of a wirelessly powered neural implant that is being developed in our research team [7,8,9]. Besides the recording and stimulation modules, it also features a controller for closed-loop operation [5,10,11], and an inductive link based Wireless Power and Data Transfer (WPDT) unit for power delivery and information transfer to/from the implant [12,13,14,15,16,17,18,19]. This unit includes an AC/DC converter, a signal modulation/demodulation stage, and a power management unit to provide regulated biasing voltages to the circuit elements of the implant. An external headstage wirelessly connects the implanted device to a computer hub for data acquisition, system monitoring, and parameters configuration [20]. Photovoltaic cells and rechargeable batteries are considered for supplying the headstage [21,22,23,24]. Within this architecture, this paper focuses on the stimulation section.

In most cases, neural stimulation uses current mode techniques to force a flow of charge through the extracellular fluid of some excitable nervous system tissue accessed with microelectrodes [5,6]. Electrical stimulation typically consists of as a series of biphasic current pulses. The amplitude and duration of both phases, called anodic and cathodic, are conveniently adjusted to result in an overall zero net charge in the tissue. Important factors in the design of neural stimulators are the characteristics of the electrode–tissue interface (ETI), which depends on the geometry and materials of the electrodes and can change throughout the life cycle of the implant, the physiological parameters of the tissue, and the degree of electrical contact at the stimulation zone [25].

With stimulation currents ranging in practice from a few tens of μA to a few mA and considering the wide range of ETI load impedances, the voltage compliance required by a neural stimulator can vary from a few volts to more than 10 V [26,27,28,29]. This requires programmable structures in which both the current-driving capabilities and supply voltage can be adjusted to minimize power losses at the stimulation node. This is particularly compelling in implanted devices that are supplied by remote powering techniques, as in the example of Figure 1.

In this work, a high-compliance, programmable fully on-chip neural stimulator is proposed. Instead of using an HV CMOS node [30,31], the whole system is designed in a standard 1.8 V/3.3 V 0.18 μm CMOS process to enable the implementation of a single SoC implant, along with other elements already designed in this technology [9]. To do so, circuit solutions are implemented to ensure voltage drops across devices are below breakdown limits, even with compliance voltages well above the technology’s nominal supply. Previous contributions have also addressed the design of a HV neural stimulator in a LV CMOS process [28,29,32,33]. However, these solutions present some drawbacks such as limited power supply range and moderate current mismatch [33], low efficiency [28], lack of control in the stimulation charge [32], and fixed power supply [29]. These aspects are addressed in this work, where the proposed neural stimulator can handle voltage supplies up to 4× higher than the technology’s nominal supply and stimulation currents up to 2 mA; the residual charge is below 0.12% for the whole stimulation range, achieving 48% power efficiency at a maximum stimulation current.

The paper is organised as follows. In Section 2, the operation of a current-controlled biphasic stimulator is described, with emphasis on the analysis of the compliance voltage and power efficiency. The architecture, components, and operation modes of the proposed neural stimulator are described in Section 3. In Section 4, experimental results are presented and discussed. Finally, Section 5 concludes the paper with some remarks.

## 2. Current-Mode Biphasic Stimulation: Compliance Voltage and Power Efficiency

**Figure 2:**
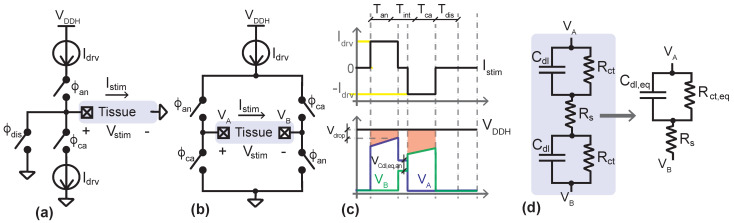
(**a**,**b**) Simplified schematics of unipolar and bipolar electrical neural stimulators. (**c**) Current and voltage stimulation waveforms. (**d**) Electrical model of the ETI and lumped model.

Figure 2a,b show the simplified schematics of a monopolar neural stimulator and a bipolar neural stimulator, respectively. They essentially work in two alternating phases. First, an anodic phase of $T_{an}$ duration is established to inject charge immediately around the stimulation electrode A. Then, a cathodic phase of $T_{ca}$ duration is set to restore the charge balance in the tissue prior to the stimulation. This is done to prevent charge accumulations that can lead to the generation of toxic chemicals or the corrosion of the electrodes [25]. Hence, if $I_{stim,an}$ and $I_{stim,ca}$ are the currents flowing through the ETI during the anodic and cathodic phases, the charges $Q_{an}=\int_{0}^{T_{an}}I_{stim,an}\;dt$ and $Q_{ca}=\int_{0}^{T_{ca}}I_{stim,ca}\;dt$, injected and extracted from the tissue, respectively, should have the same magnitude but a different sign. Generally, the stimulation currents during the anodic and cathodic phases may have different waveforms and a duration as long as $|Q_{an}\left|=\right|Q_{ca}|$; however, in this work we consider a typical case in which such currents are pulses of the same duration, $T_{an}=T_{ca}$, and the same magnitude, $|I_{stim,an}\left|=\right|I_{stim,ca}|=I_{drv}$.

It has been experimentally demonstrated that the use of a cathodic phase to reverse the electrochemical process occurring in the anodic phase may eventually preclude the generation of action potentials [25]. To prevent this suppressing effect, an interphase delay, $T_{int}$, of around 100 μs is typically introduced in the biphasic stimulation. Considering this delay, the complete stimulation waveform is illustrated in Figure 2c.

The stimulation mechanism differs depending on the circuit topology. In the monopolar case, two different current sources are used to inject and sink a charge from a single stimulation node A, while in the bipolar case a single current source is used for both purposes at the expense of using two electrodes, A and B. In this latter case, the anodic current flows from node A to B, while in the cathodic phase, the current flows in the reverse direction, from B to A. In both circuits, the interphase delay is simply implemented by opening all the switches between the anodic and cathodic phases. In this state, there is no current flow, the electrical potential across the ETI is constant, and the electrode voltages take values between the power supply of the neural stimulator, $V_{DDH}$, and ground. These values are determined by voltage division according to the impedance of the switches in the same circuit branch.

Due to the non-stationary nature of the ETI, its nonlinear characteristics, and the fact that some electrochemical reactions are not reversible, circuit techniques to guarantee the desired charge balance between the anodic and cathodic phases are needed to improve the safety of the stimulator. In the case of monopolar stimulation, charge balancing is even more difficult due to the potential mismatch between the current sources used for injecting and retrieving charge from the tissue. In contrast, the bipolar topology is intrinsically tolerant to matching problems because it uses only one current source. A simple approach, suitable both for monopolar and bipolar structures, to force $|Q_{an}\left|=\right|Q_{ca}|$ even in the case of perfectly matched current sources, is to trigger a post-stimulation blanking period in which the electrodes are short-circuited to ground for a time $T_{dis}$ [25]. As shown in Figure 2a, this requires an extra switch in the monopolar topology, while in the bipolar scheme the bottom switches in Figure 2b can be reused for the discharge phase. Figure 2c also shows this blanking phase. In this work, a high-precision bipolar stimulator with discharging phase is designed.

Figure 2d shows a simplified model of the equivalent impedance, $Z_{L}$, between the stimulation nodes A and B in the schematics of Figure 2b [25,34]. Such impedance is given by

(1) $$Z_{L}\left(s\right)=R_{s}+\frac{R_{ct,eq}}{1+s\;\cdot \;R_{ct,eq}\;\cdot \;C_{dl,eq}},$$

where $R_{s}$ models the spreading resistance of the neural tissue, $C_{dl,eq}=C_{dl}/2$ takes into account the electrical double-layer capacitance at the ETI, and $R_{ct,eq}=2R_{ct}$ is an equivalent charge transfer resistance that models the faradaic electrochemical reactions at the electrode surface [25]. The voltage between the electrodes A and B, $V_{stim}\left(t\right)=\left|V_{A}\left(t\right)-V_{B}\left(t\right)\right|$, during the anodic phase (a similar analysis can be done for the cathodic phase) is given by

(2) $$V_{stim}\left(t\right)=I_{drv}\;\cdot \;R_{s}+V_{C_{dl,eq}}\left(t\right),$$

where $t\in [0,T_{an}]$ and $V_{C_{dl,eq}}\left(t\right)$ is the voltage across the equivalent double-layer capacitance given by

(3) $$V_{C_{dl,eq}}\left(t\right)=I_{drv}\;\cdot \;R_{ct,eq}-\left(I_{drv}\;\cdot \;R_{ct,eq}+V_{C_{dl,eq,0}}\right)\;\cdot \;e^{-\frac{t}{\tau }},$$

where $V_{C_{dl,eq,0}}$ is the $V_{C_{dl}}$ voltage stored at the beginning of the pulse and $\tau =R_{ct,eq}\;\cdot \;C_{dl,eq}$. Assuming that the time constant of the ETI is much larger than the duration of the anodic phase, i.e., $T_{an}\ll \tau$ as it occurs in practice, the peak stimulation voltage between the electrodes at the end of the anodic phase ($t=T_{an}$) (preserved during the interphase delay period) can be approximated as

(4) $$V_{stim,pk}\approx V_{C_{dl,eq,0}}+I_{drv}\left(R_{s}+\frac{T_{an}}{C_{dl,eq}}\right).$$

The efficiency $\eta_{stim}$ of the neural stimulator during the anodic phase can be defined by the ratio between the energy delivered to the tissue and the energy supplied by the voltage supply, $V_{DDH}$. Hence, assuming again that $T_{an}\ll \tau$, the following expression is obtained:

(5) $$\eta_{stim}=\frac{\int_{0}^{T_{an}}V_{stim}\left(t\right)\;\cdot \;I_{drv}\;\cdot \;dt}{\int_{0}^{T_{an}}V_{DDH}\;\cdot \;I_{drv}\;\cdot \;dt}\approx \frac{I_{drv}}{V_{DDH}}\;\cdot \;\left(R_{s}+\frac{T_{an}}{2\;\cdot \;C_{dl,eq}}\right),$$

which shows that the efficiency depends on the load impedance and the pulse characteristics. Clearly, the efficiency increases by reducing the supply voltage $V_{DDH}$ up to the limit imposed by the peak stimulation voltage $V_{stim,pk}$ in (4). On the other hand, for a given $V_{DDH}$ value, the efficiency and peak of the stimulation voltage decrease both with the amplitude and width of the current pulse, and the stimulator may be forced to withstand a large voltage gap between the supply voltage and $V_{A}$. These considerations are taken into account in the proposed design.

## 3. System Architecture and Circuit Design

**Figure 3:**
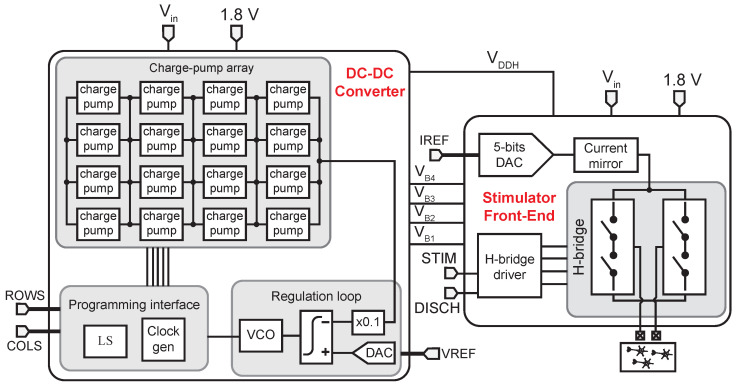
Block-level scheme of the proposed HV neural stimulator.

Figure 3 shows the proposed HV electrical neural stimulator. It consists of two main blocks: a fully on-chip power management unit and a stimulator front-end. The former includes a DC-DC converter, which provides a programmable supply voltage $V_{DDH}\in \left[4.2,13.2\right]$ V for the stimulation driver; a resistorless bandgap [35]; and a 25 nA self-biased current reference [36] (not shown in Figure 3 for simplicity). The stimulator front-end comprises a 5 bits current-steering digital-to-analog converter (DAC), a current mirror that generates a current $I_{drv}\in \left[0.07,2.08\right]$ mA, a high-voltage-tolerant H-bridge biphasic stimulation interface, and an H-bridge driver. This latter includes a high-compliance voltage cell (HCVC) to protect the circuit from excessive voltage drops between the transistor’s terminals.

### 3.1. Switched-Capacitor DC-DC Converter

As shown in Figure 3, it is based on a 4 × 4 array of individually configurable charge pump (CP) cells. The outputs of all the CPs in the same column are connected together. Active rows and columns are enabled using the $\mathrm{ROWS}=\{ROWS_{i}\},i=1,\ldots ,4,$ and $\mathrm{COLS}=\{COLS_{j}\},j=1,\ldots ,4,$ configuration words, respectively. All possible row combinations, 16 in total, are possible. However, a column can only be activated if the previous one is enabled and, therefore, only four combinations are possible. In the following, the number of activated rows (alt. columns) will be denoted as $M_{a}$ (alt. $N_{a}$).

The output voltage $V_{DDH}$ of the converter is locked by a regulation loop that sets the pumping frequency, $f_{p}$, of the CP cells. This frequency ranges from approximately 5 MHz to 60 MHz. Under lock-in conditions, $V_{DD,H}=10\;\times \;V_{ref}$ for $V_{ref}\in \left[0.42,1.32\right]$, and is defined as

(6) $$V_{DDH}=N_{a}\;\cdot \;V_{pump}+V_{in},$$

where $V_{in}$ is the input voltage of the CP cells (3 V in this work) and $V_{pump}$ is the voltage pumped by each stage in an enabled column. This voltage is given by

(7) $$V_{pump}=V_{in}-R_{CP}\;\cdot \;I_{L},$$

where $I_{L}$ is the load current of the DC-DC converter and $R_{CP}$ is the equivalent resistance of the CP cell given by

(8) $$R_{CP}=\frac{N_{a}}{2\;\cdot \;M_{a}\;\cdot \;f_{p}\;\cdot \;C_{fly}},$$

where $C_{fly}$ is the flying capacitance of each charge pump cell [37]. In this design, $C_{fly}$ amounts to 12.5 pF.

**Figure 4:**
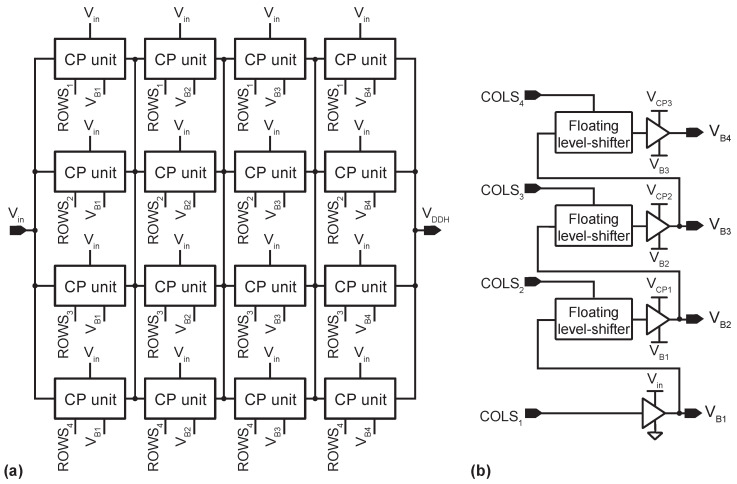
(**a**) Block diagram of the CP array. (**b**) Schematic of the circuit generating the voltage-shifted versions of the column selection signals, $COLS_{i}$. $V_{CPi}$ corresponds to the output voltage if the *i-th* column of the CP array.

Programming the number of active columns requires the DC-DC converter to internally generate four bias voltages $V_{B1-B4}$, which are voltage-shifted versions of the selection signals, $COLS_{1-4}$. Table 1 shows the expressions for $V_{B1-B4}$ in terms of $N_{a}$, and Figure 4a illustrates the connection between the CP units and the distribution of the control signals $ROWS_{i}$ and $V_{Bj}$. Figure 4b shows the circuit that generates the bias voltages $V_{Bj}$ (by construction, $COLS_{1}=V_{B,1}=V_{in}$). The blocks that slide the signals $COLS_{j}$ are floating level-shifters (FLS) [38,39]. These circuits can tolerate non-periodical input signals and shifting voltage variations and are suitable for non-HV technology processes—see [39] for more details.

**Figure 5:**
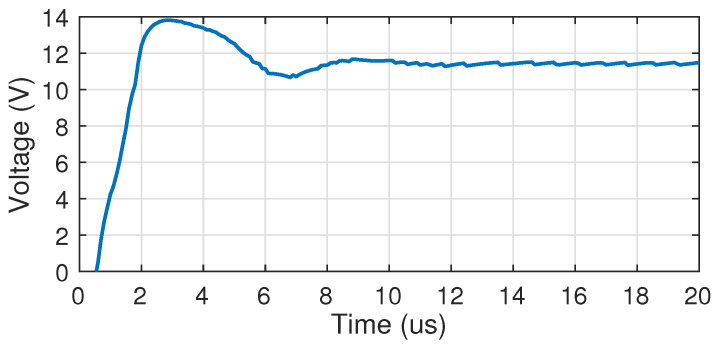
Simulation of the startup of the DC-DC converter. The output settles at a 11.5 V output voltage in roughly 12 μs.

Figure 5 illustrates the operation of the DC-DC converter at startup for a target operation point with $V_{DDH}=11.5$ V, $I_{L}=1$ mA, $M_{a}=3$, and $N_{a}=4$. As can be seen, the desired output voltage is reached after roughly 12 μs.

### 3.2. H-Bridge

**Figure 6:**
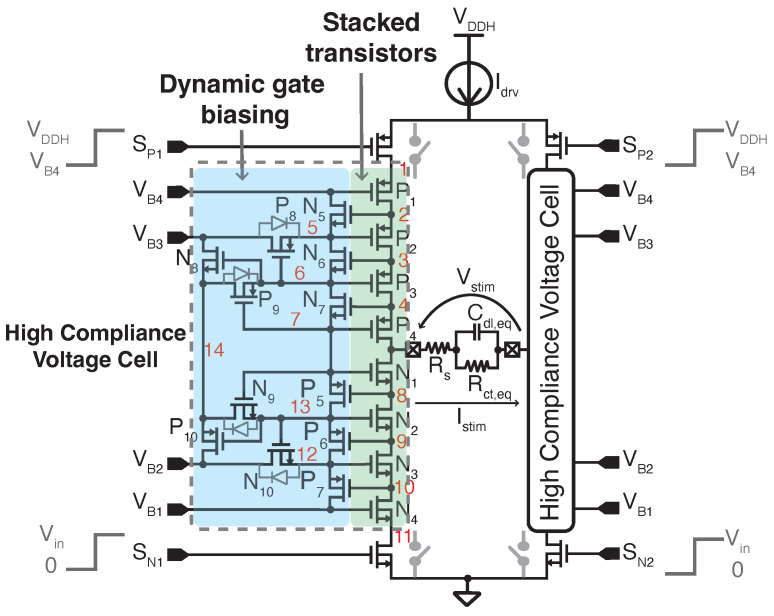
Proposed high-voltage-tolerant H-bridge, including the HCVC.

Figure 6 shows the proposed high-voltage-tolerant H-bridge. Each branch has one PMOS and one NMOS switch (driven by signals $S_{Px}$ and $S_{Nx}$, respectively) as well as a HCVC. This circuit consists of eight stacked transistors (shaded in green) and a dynamic gate biasing circuit [40,41,42] (shaded in blue). NMOS transistors are implemented in Deep N-Wells, whereas PMOS transistors have local N-Wells. The proposed solution follows the design principles presented in [42] but includes modifications aimed at extending the operation range. Modifications consist of two additional stacked transistors and four additional transistors in the dynamic gate biasing circuit. Moreover, the HCVC is biased with the voltages internally generated by the DC-DC converter $V_{B,1-4}$, and, hence, no additional dynamic biasing circuitry is needed. The circuit allows one to power the front-end of the stimulator with a wide range of voltages (up to 4 times the nominal voltage of the technology) while driving a wide range of ETI impedances and without damaging the 3.3 V stacked transistors.

To describe the operation of the proposed high-voltage-tolerant H-bridge, two simulations are carried out. In both cases, the load is purely resistive and $S_{P1}=V_{B4}$, $S_{P2}=V_{DDH}$, $S_{N1}=0$, and $S_{N2}=V_{in}$. Thus, the top-left and bottom-right switches are ON, whereas the top-right and bottom-left switches are OFF.

**Figure 7:**
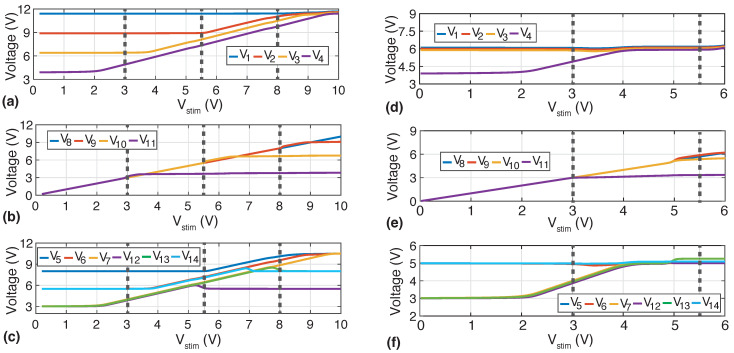
Evolution of the voltages at the nodes of the HCVC, as depicted in Figure 6. (**a**–**c**) The load resistance, $R_{s}$, is swept from 10 Ω to 5 kΩ, $V_{DDH}$ = 13 V, and $N_{a}\;=\;4$. (**d**–**f**) The load resistance, $R_{s}$, is swept from 0 to 3 kΩ, $V_{DDH}$ = 8 V, and $N_{a}\;=\;2$.

In the first simulation, the voltage supply of the neural stimulator front-end is set to 13 V, $I_{drv}=2$ mA; $R_{s}$ is swept from 10 Ω to 5 kΩ; and $N_{a}=4$ (i.e., $V_{B1}=3$ V, $V_{B2}=5.5$ V, $V_{B3}=8$ V, and $V_{B4}=10.5$ V—see Table 1). As shown in Figure 7a, when $V_{stim}<V_{in}$, transistors $P_{1-4}$ are saturated and equally withstand the voltage drop across the H-bridge. As $V_{stim}$ increases, the voltage $V_{4}$ also increases, so transistor $N_{7}$ turns on, $V_{7}$ approaches $V_{6}$, and transistor $P_{4}$ enters in triode mode. As $V_{stim}$ increases further, this behavior is sequentially repeated for voltage $V_{3}$, transistor $N_{6}$, voltage $V_{6}$, and transistor $P_{3}$, respectively, and then for voltage $V_{2}$, transistor $N_{5}$, voltage $V_{B4}$, and transistor $P_{2}$. Transistors $P_{8}$ (resp. $P_{9}$) ensure that nodes $V_{5}$ (resp. $V_{6}$) are gradually connected to $V_{B3}$. Moreover, as $V_{stim}$ increases, transistor $N_{8}$ starts to turn on and continuously connects node 14 to $V_{B3}$.

On the contrary, as shown in Figure 7b, when $V_{stim}<V_{in}$, transistors $N_{1-4}$ act as closed switches, i.e., they are biased in the deep triode region. As $V_{stim}$ rises, so do $V_{8-11}$, thus switching $P_{5-7}$ off. At the same time, $N_{9-10}$ progressively make cause $V_{12,13}$ to approach $V_{B2,14}$, respectively. Thus, $N_{1-4}$ gradually increase their drain-to-source voltage to a maximum drop of roughly $V_{in}$. Finally, opposite $N_{8}$, transistor $P_{10}$ starts biasing node 14 to $V_{B2}$ and continuously switches off. The gate voltages of the stacked transistors, which permit proper biasing to adapt the stimulation voltage, are shown in Figure 7c.

In the second simulation, the voltage supply of the neural stimulator front-end is set to 7 V, $N_{a}=2$. (i.e., $V_{B1}=3$ V, $V_{B2}=5$ V, $V_{B3}=5$ V, and $V_{B4}=5$ V), and $R_{S}=\left[0,3\right]$ kΩ. In this case, as shown in Figure 7d, when $V_{stim}<V_{in}$, transistors $P_{1-3}$ are biased in deep triode region, whereas $P_{4}$ is saturated. Thus, now the voltage drop in the HCVC is approximately the source-to-drain voltage of $P_{4}$, which is around 2 V. As $V_{stim}$ increases, $P_{4}$ gradually enters in deep triode region, leading to a voltage drop across the HCVC close to zero. Figure 7e shows that transistors $N_{1-4}$ are biased in the deep triode region and they sequentially enter in saturation as $V_{stim}$ rises. As before, Figure 7f shows the gate voltages of the stacked transistors.

The simulations in Figure 7 show that the proposed H-bridge has two fundamental features. First, it withstands up to $4\times V_{DD}$ voltage differences between input output nodes. Second, it acts as an H-bridge with low on resistance. Both behaviors manifest depending on the stimulation current, the supply voltage $V_{DDH}$, and the load impedance.

### 3.3. DAC and Current Mirror

**Figure 8:**
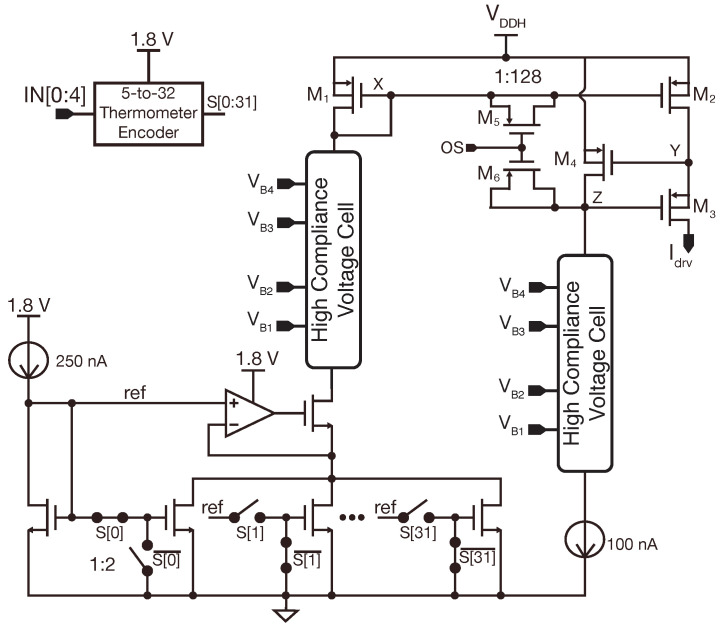
Schematic of the 5 bits current-steering DAC, current mirror, and two HCVCs for interfacing LV and HV circuits. Biasing currents are copies of an on-chip 25 nA self-biased current source [36].

Figure 8 shows the schematics of the DAC and current mirror represented in Figure 3.

The DAC uses a 5-bit thermometric current-steering topology and is supplied at 1.8 V. A MUX-based 5-to-32 thermometer decoder [43] is used for converting the binary input to thermometric. The output impedance of the DAC is enhanced by means of a regulated cascode topology with a current-mirror OTA. The DAC output current can reach up to 15.6 μA at 0.5 μA steps.

The current mirror is supplied at $V_{DDH}$, it has a gain of 128; and it implements a regulated cascode current mirror topology that achieves good output impedance, a fast transient response, and uses no operational amplifiers [44]. Two MOS capacitors of approximately 110 fF are added to reduce overshoots in the output current during the anodic and cathodic phases. Two HCVC cells connect the DAC output and the biasing current of the regulated cascode circuit to the current mirror.

### 3.4. H-Bridge Driver

**Figure 9:**
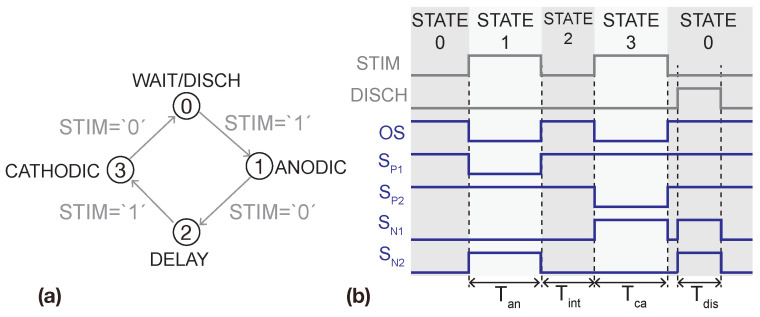
(**a**) Mealy’s state machine implemented for driving the H-bridge. (**b**) Timing diagram of signals generated by the H-bridge driver.

The H-bridge driver converts the input signals *STIM* and *DISCH* to the four signals driving the H-bridge—$S_{P,1-2}$ and $S_{N,1-2}$—and to the signal reducing the current overshoot, *OS*. Figure 9 shows a timing diagram of these signals. It consists of a Mealy’s finite state machine and level shifters for adapting the signals to the adequate voltage level. The level shifters driving the PMOS switches of the H-bridge are implemented as FLS.

## 4. Experimental Results

**Figure 10:**
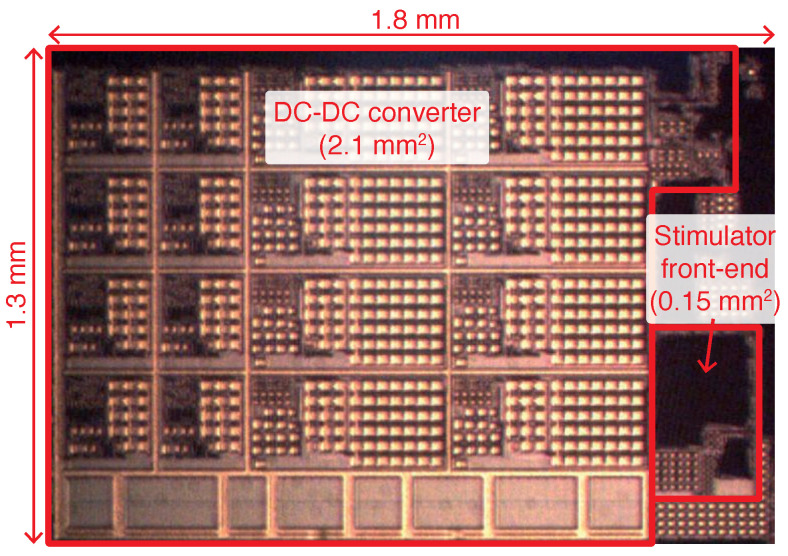
Microphotograph of the fabricated chip.

Figure 10 shows a micro-photograph of the proposed neural stimulator, fabricated in a standard 0.18 μm 1.8 V/3.3 V CMOS process. The circuit occupies an active area of 2.34 ${\mathrm{mm}}^{2}$, including the on-chip switched-capacitor DC-DC converter (2.1 ${\mathrm{mm}}^{2}$), the stimulator front-end 0.15 ${\mathrm{mm}}^{2}$), the internal serial peripheral interface (SPI) module for communication, and other test circuitry. No external components are needed.

### 4.1. Electrical Characterization

**Figure 11:**
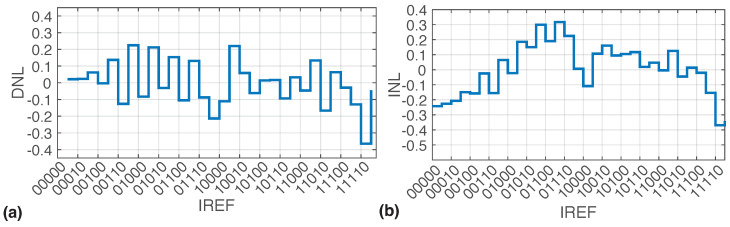
Stimulation current’s (**a**) DNL and (**b**) INL. Both were measured for a 4.7 kΩ load and $V_{DDH}$ = 11 V and are represented as a fraction of one LSB.

Electrical characterization was carried out using a test PCB in which a load impedance $Z_{L}$ with values $R_{s}$ = 4.7 kΩ, $C_{dl,eq}$ = 330 nF, and $R_{ct,eq}$ = 40 MΩ was mounted [29,34]. Figure 11a,b show the measured differential non-linearity (DNL) and integral non-linearity (INL) of the neural stimulator. INL was calculated as the deviation of the response from the best-fit straight line [45]. It can deliver currents from 69 μA up to 2.08 mA with a least-significant bit (LSB) current of approximately $I_{LSB}$ = 65 μA. Given that $V_{DDH}$ reaches 13.2 V, the stimulator can deliver a current of 2.08 mA to resistive loads close to 6.3 kΩ.

**Figure 12:**
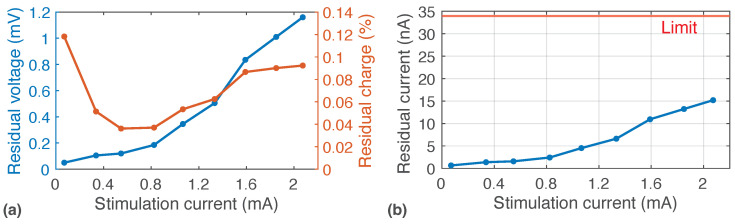
(**a**) Measured residual voltage and residual charge stored at $C_{dl}$ after biphasic stimulation rounds with no discharging phase. $T_{an/ca}$ = $T_{int}$ = 200 μs. (**b**) Average residual current, as defined in [29].

Figure 12a depicts the residual voltage and residual charge stored at the double-layer capacitance for different stimulation currents. The residual voltage was measured by delivering 200 biphasic stimulation rounds with anodic/cathodic and interphase phases lasting 200 μs. No discharging phase was triggered. Voltage at $C_{dl,eq}$ was sampled and converted with an ADC before the first stimulation round and after the 200-th stimulation round, then it was averaged. The residual voltage remains below 1 mV for most of the stimulation current range. According to the criteria in ISO 14708-1, the average residual current—defined as the residual charge at the ETI divided by the time between stimulation rounds [29]—should be below 0.75 μA ${\mathrm{mm}}^{-2}$ [29,46]. Considering a 120 μm electrode, this limit is calculated as 34 nA. Figure 12b depicts the measured average residual current, as calculated in [29]. It is shown that the measured residual current after a single biphasic stimulation round is below the limit for the whole stimulation range. Expressed as a percentage of the charge delivered during each stimulation phase, the residual charge is less than 0.1% for most of the stimulation current range.

**Figure 13:**
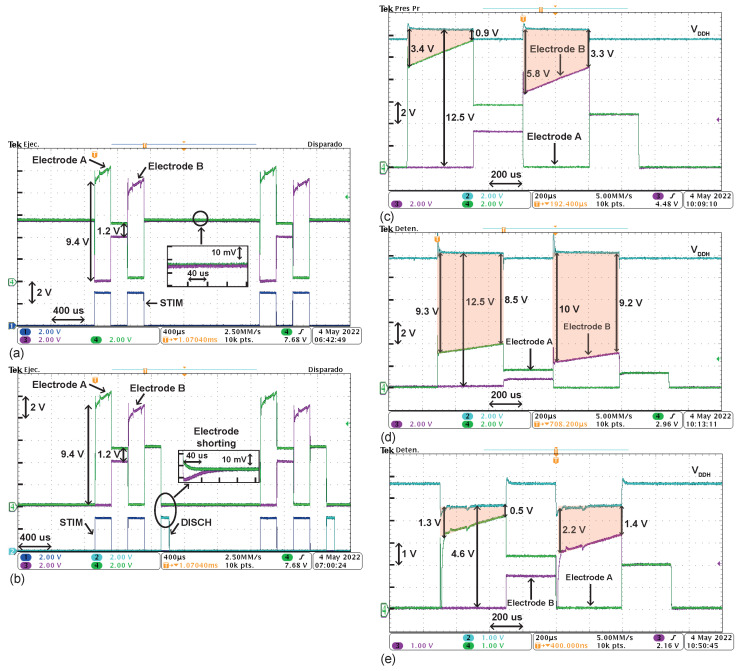
(**a**,**b**) Electrodes’ voltages when a 2 mA stimulation current is delivered with/without electrode shorting phase. $T_{an/ca}$ = $T_{int}$ = 200 μs and $V_{DDH}$ = 12 V. (**c**–**e**) Electrodes’ voltages and DC-DC converter output voltage at different stimulation timing and currents. Voltage drop between $V_{DDH}$ and stimulator’s output is shaded in red.

Figure 13a,b illustrate the use of the discharging phase by electrode shorting to remove the residual voltage. A 2 mA current is delivered with $T_{an/ca}$ = $T_{int}$ = 200 μs and $V_{DDH}$ = 12 V.

On the one hand, Figure 13a shows both electrodes’ voltage when no discharging phase is triggered. The 2 mA current causes an instant 9.4 V difference between electrodes when flowing through $R_{s}$ = 4.7 kΩ. The voltage at electrode A reaches around 10.6 V. Moreover, after the anodic phase, the voltage stored at $C_{dl,eq}$ = 330 nF is 1.2 V, as expected by observing (3). In the cathodic phase, the voltage at node B reaches roughly 9.4 V. Then, after the biphasic stimulation, a fraction of the charge stored at the parasitic capacitance seen by node B flows to the parasitic capacitance seen by node A, thus causing a non-zero absolute voltage in both nodes. Due to the negligible size of parasitic capacitances at nodes A and B, this effect, also seen in other reported works [30,33], does not have a meaningful impact on the operation of the stimulator. On this point, the voltage difference between both outputs is in the range of milli-volts, as shown in Figure 12. Hence, even with the zoomed screenshot, the residual voltage can not be accurately measured.

On the other hand, Figure 13b shows both electrodes’ voltage when a 100 μs electrode shorting phase is triggered, which discharges both electrodes to ground. A zoom of the electrode shorting phase is also included. After the biphasic pulses, the stimulator outputs’ parasitic capacitances shared their stored charges again. When applying the electrode shorting, both outputs were discharged to ground. The zoomed screenshot is provided as a qualitative graphical description of the discharging phase. However, the perturbations introduced by oscilloscope’s probes do not allow one to obtain accurate quantitative data in the millisecond-amplitude and microsecond-time scales of the residual voltage nor the discharging time.

Figure 13c–e depict the electrodes’ voltages and DC-DC converter’s output voltage, $V_{DDH}$, in different scenarios. The area of regions shaded in red multiplied by the stimulation current represents energy losses at the stimulator front-end, as discussed in Figure 2. Figure 13c shows how the system handles the delivery of a stimulation current of roughly 2 mA to the load, with $V_{DDH}$ = 12.5 V. The voltage at electrode A goes from 9.1 V to 11.6 V. The stimulator front-end is thus capable of delivering a high stimulation current with a dropout voltage below 1 V while being supplied at a voltage four times higher than the nominal voltage supply of the technology.

Figure 13d shows how the system handles the delivery of a stimulation current of roughly 0.7 mA to the load, with $V_{DDH}$ = 12.5 V. In this case, there is a large voltage drop from $V_{DDH}$ to the electrodes. However, as discussed in Section 3 the HCVC maintains the voltage across all devices below 3.3 V.

Figure 13e illustrates how the programmability of $V_{DDH}$ improved the power efficiency. The response of the system was measured again with $I_{drv}$ = 0.7 mA, but $V_{DDH}$ was now set to 4.6 V. With this current level, the neural stimulator can operate with a voltage drop of 0.5 V.

**Figure 14:**
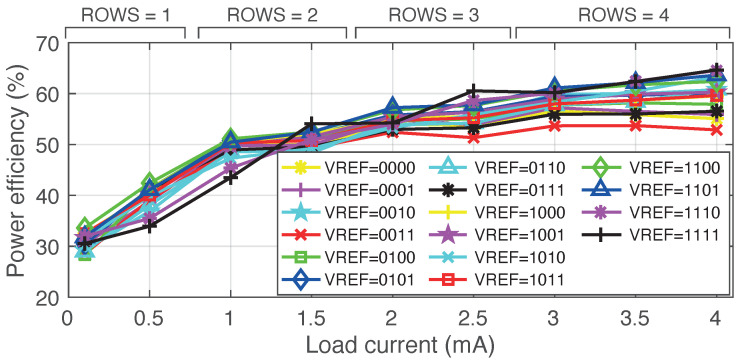
Measured DC-DC converter’s power efficiency for different load currents.

Regarding the on-chip HV generation, Figure 14 shows DC-DC converter’s power efficiency, $\eta_{conv}$, for $V_{DDH}\in \left[4.2\;V,13.2\;V\right]$ at different load currents. The final design of the neural stimulator will include multiple cores in order to feature multi-site stimulation. Hence, the DC-DC converter was designed to deliver up to 4 mA. The 4-bit word **VREF** is used for target voltage selection. Moreover, **ROWS** are adapted to the load current as shown in the figure, whereas **COLS** are adapted to the output voltage –**COLS** = 1 for VREF∈[‘0000’,‘0010’]; **COLS** = 2 for VREF∈[‘0011’,‘0110’]; **COLS** = 3 for VREF∈[‘0111’,‘1010’]; and **COLS** = 4 for VREF∈[‘1011’,‘1111’]. Thus, the DC-DC converter’s power efficiency in the operation points shown in Figure 13c–e is 58%, 46%, and 45%, respectively. This way, the neural stimulator’s measured overall efficiency, $\eta_{stim}$, was 48% at the operation point ($V_{DDH}$, $I_{drv}$), equal to (12.5 V, 2 mA); 13% at (12.5 V, 0.7 mA); and 36% at (4.6 V, 0.7 mA). Regardless of the target **VREF**, efficiency increases monotonically with the load current; thus, it is expected to have a DC-DC converter’s power efficiency roughly from 28% up to 57% for the load current range of a single neural stimulation core.

### 4.2. Measurements on a Phosphate-Buffered Saline (PBS) Solution

**Figure 15:**
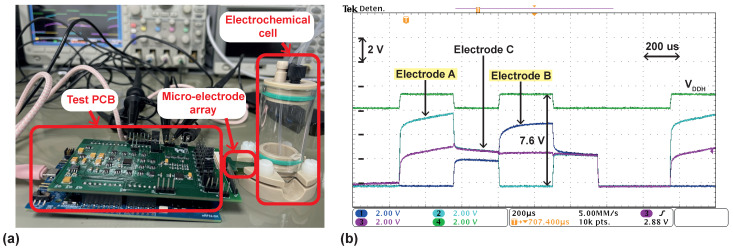
(**a**) Photography of the test-bench for stimulation in a PBS solution. (**b**) Electrodes’ voltages when a 2 mA stimulation current is delivered to a PBS solution.

The neural stimulator was also characterized by immersing a custom μ electrode array with 120 μm-diameter electrodes covered with gold into a phosphate-buffered saline (PBS) solution by means of an electrochemical cell, as depicted in Figure 15a. Figure 15b shows the response when a 2 mA stimulation current is delivered between two electrodes separated roughly 600 μm away in the custom μ electrode array (A and B in the oscilloscope screenshot). Stimulation timing was configured as $T_{an}$ = $T_{ca}$ = 300 μs, $T_{int}$ = 250 μs, and $T_{dis}$ = 200 μs. $V_{DDH}$ is set at 7.6 V during stimulation and decreased to roughly 6.6 V between stimulation phases. From the curves of voltages at electrodes A and B, it can be seen that the response of the electrodes immersed in the PBS solution approaches a series resistance-capacitance circuit with $R_{s}\approx 2.3\;k\Omega$ and $C_{dl,eq}\approx 550\;\mathrm{nF}$. Electrode C, also shown in Figure 15b, is located in the vicinity of electrodes A and B.

### 4.3. State-of-the-Art Comparison

Table 2 summarizes the performance of the proposed neural stimulator, along with other solutions proposed in the literature. Compared to the reported HV systems implemented in LV CMOS processes, the proposed neural stimulator achieves higher compliance voltage and wider $V_{DDH}$ than any other reported solution. It also achieves lower charge mismatch when no charge balancing phase is triggered than other works [29,33]. Moreover, a lower area/channel than [28,33] was achieved. Finally, when delivering 2 mA of current, similar power efficiency as in [33] was obtained, whereas the 36% power efficiency obtained at 0.7 mA stimulation current outperformed that reported in the mentioned work.

## 5. Conclusions

This paper reports a fully integrated HV neural stimulator, which intends to be implemented in a wirelessly powered neural implant. The on-chip switched-capacitor DC-DC converter generates the stimulator front-end’s voltage supply, $V_{DDH}\in \left[4.2,13.2\right]$ V. The stimulator front-end can deliver a wide range of stimulation currents, up to 2 mA at roughly 65 μA steps, making it suitable for quite diverse stimulation scenarios, both in rodents and mammals.

Implemented in a standard 1.8 V/3.3 V CMOS process, it can handle voltages up to four times higher than the nominal process supply while keeping device terminal voltages below safe limits, thus ensuring long-term reliability. This is accomplished by implementing circuits such as a novel HCVC, which adapts its equivalent impedance in order to withstand high voltages or act as a closed switch, as needed.

Power efficiency can be maximized by adapting the programmable output voltage of the DC-DC converter to minimize $V_{drop}$, achieving 48% efficiency for a stimulation current of 2 mA and $V_{DDH}$ = 11.6 V. In this regard, the single-chip neuromodulator core that is being developed will include a feedback loop to monitor electrodes’ voltages and adjust $V_{DDH}$ accordingly.

## Figures and Tables

**Table 1:** DC-DC converter’s output voltage and biasing voltages, depending on the number of stages enabled. $V_{pump}$ is defined in (7).

Node	$N_{a}=1$	$N_{a}=2$	$N_{a}=3$	$N_{a}=4$
$V_{DDH}$	$V_{in}+V_{pump}$	$V_{in}+2\;\cdot \;V_{pump}$	$V_{in}+3\;\cdot \;V_{pump}$	$V_{in}+4\;\cdot \;V_{pump}$
$V_{B1}$	$V_{in}$	$V_{in}$	$V_{in}$	$V_{in}$
$V_{B2}$	$V_{in}$	$V_{in}+V_{pump}$	$V_{in}+V_{pump}$	$V_{in}+V_{pump}$
$V_{B3}$	$V_{in}$	$V_{in}+V_{pump}$	$V_{in}+2\;\cdot \;V_{pump}$	$V_{in}+2\;\cdot \;V_{pump}$
$V_{B4}$	$V_{in}$	$V_{in}+V_{pump}$	$V_{in}+2\;\cdot \;V_{pump}$	$V_{in}+3\;\cdot \;V_{pump}$

**Table 2:** Performance comparison with previously reported works.

	[33]	[30]	[28]	[31]	[29]	This Work
CMOS Process	0.18 μm 1.8 V/3.3 V	0.18 μm 24 V	65 nm LV	0.25 μm 2.5 V/5 V/12 V	0.18 μm 1.8 V/3.3 V	0.18 μm **1.8 V/3.3 V**
$V_{DDH}$ gen.	6.7–12.3 V (4b)	Up to 22.5 V	11 V	20 V	±6 V	**4.2–13.2 V (4b)**
$V_{DDH}$ gen. area	1.5 mm^2^	Off-chip	0.04 mm^2^	1.64 mm^2^	-	2.1 mm^2^
Stim. current	2.48 mA (5b)	48.4 μA (7b) 169.5 μA (7b)	2 mA (8b)	5 mA (6b)	3 mA (4b)	**2.08 mA (5b)**
Area/channel	1.5 mm^2^	-	0.36 mm^2^	0.22 mm^2^	0.08 mm^2^	0.15 mm^2^
Compliance voltage	11 V	21.3 V	11 V	16.7 V	3.6 V	**12.5 V**
Max. $Q_{res}/Q_{stim}$	1.7%	0.03%	-	-	1.94%	**0.12%**
Power efficiency	48% (2 mA, 10 V) 32% (1 mA, 6.7 V)	-	31% (2 mA) 28% (1 mA)	-	-	**48% (2 mA, 11.6 V)** **36% (0.7 mA, 4.6 V)**

## Data Availability

Not applicable.

## Abbreviations

The following abbreviations are used in this manuscript:

HV	high-voltage
LV	low-voltage
ETI	electrode–tissue interface
BLE	Bluetooth Low Energy
WPDT	Wireless Power and Data Transfer
SoC	System on Chip
HCVC	high-compliance voltage cell
DAC	digital-to-analog converter
FLS	floating level-shifters
DNL	differential non-linearity
INL	integral non-linearity
LSB	least-significant bit
SPI	serial peripheral interface
